# Antibiotic resistance and the COVID‐19 pandemic: A dual crisis with complex challenges in LMICs

**DOI:** 10.1002/hsr2.1566

**Published:** 2023-09-13

**Authors:** Bashar Haruna Gulumbe, Muhammed Rabiu Sahal, Abdulrakib Abdulrahim, Abdullahi Adamu Faggo, Zaharadeen Muhammad Yusuf, Kabir Hassan Sambo, Nazeef Idris Usman, Musbahu Abdullahi Bagwai, Wada Nafiu Muhammad, Aliyu Adamu, Uzairu Aminu, Munkaila Tirmizhi Abubakar, Kadai Alhaji Lawan

**Affiliations:** ^1^ Department of Microbiology, Faculty of Science Federal University Birnin Kebbi Birnin Kebbi Nigeria; ^2^ Department of Biological Sciences Abubakar Tafawa Balewa University Bauchi Bauchi Bauchi State Nigeria; ^3^ Department of Microbiology Bauchi State University Gadau Bauchi State Nigeria; ^4^ Department of Biochemistry Al‐Qalam University Katsina Katsina Katsina State Nigeria; ^5^ Department of Life Sciences, School of Technology Kano State Polytechnic Kano Kano State Nigeria; ^6^ Department of Laboratory Technology Federal Polytechnic Bauchi Bauchi Bauchi State Nigeria; ^7^ Department of Microbiology and Immunology, Faculty of Biomedical Sciences Kampala International University Kampala Uganda

**Keywords:** AMR, antibiotic resistance, COVID‐19, infectious diseases, LMICs

## Abstract

**Background and Aims:**

Antimicrobial resistance (AMR), a global health crisis of mounting urgency, has been further complicated by the ongoing COVID‐19 pandemic. The intricate relationship between these two phenomena is especially pronounced in low‐ and middle‐income countries (LMICs) due to the distinct obstacles encountered by their healthcare systems and policy structures. This study aims to explore the complex challenges arising from the coexistence of these two crises in LMICs and proffer specific recommendations for holistic management.

**Methods:**

An exhaustive bibliographic survey was executed, employing search queries in specialized databases such as PubMed, SCOPUS, and Web of Science's SCI‐EXPANDED index. The timeframe for the literature search extended from January 2020 to January 2023. The search strategy employed key terms including antibiotic resistance, AMR, COVID‐19 pandemic, low‐ and middle‐income countries, SARS‐CoV‐2, and LMICs.

**Results:**

The pandemic has aggravated various drivers of AMR in LMICs, including limited capabilities, weak frameworks, and socioeconomic factors. New challenges have emerged, such as disruptions in the antibiotic supply chain and an increased risk of healthcare‐associated infections. The interaction between these drivers presents a complex problem that demands a coordinated response. Specific recommendations include strengthening health systems, funding research and innovation, and enhancing infection prevention control measures.

**Conclusion:**

The coexistence of AMR and the COVID‐19 pandemic in LMICs demands an integrated approach involving multiple stakeholders. Emphasis must be placed on constructing aligned regulatory frameworks, nurturing regional collaborations, and focusing on accessible therapeutic options. The study underscores the necessity for actionable strategies to achieve sustainable access to clean water and sanitation and also highlights the importance of long‐term planning, funding, and specialized expertise in emerging modalities like phage therapy.

## 1 INTRODUCTION

Antibiotics have transformed how infectious diseases are treated. Antimicrobial resistance (AMR), however, is growing and threatening to undermine the efficiency of these medications.^1^ This problem has been connected to the inappropriate use of antibiotics. AMR can bring about increased morbidity, mortality, healthcare expenditures, and financial losses.^2^ According to the World Health Organization (WHO), at least 700,000 people every year pass away from drug‐resistant diseases, and if immediate action is not taken to address the issue, this figure could rise to 10 million by 2050.^3^ Ninety percent of those deaths have been predicted to happen in low‐ and middle‐income countries (LMICs) with a possible cost of up to US$1 trillion.^4^ Recent research suggests a strong interplay between COVID‐19 and AMR.^4^, ^5^

The emergence of COVID‐19, which originated in China, has rapidly permeated 213 countries, including LMICs, has been described as one of the most devastating global pandemics.^6^, ^7^ As of August 2nd, 2023, the pandemic has claimed 6,953,743 lives and resulted in 768,983,095 confirmed cases worldwide, according to the WHO's dashboard.^8^ The extensive impact of the virus underscores the urgent need for continued vigilance and coordinated response. The containment of COVID‐19 in LIMCS has been challenging due to limited resources, inadequate healthcare providers, lack of personal protection equipment and serious issues related to healthcare system which has been exacerbated by the pandemic.^9^, ^10^ More worrisome is the possibility of COVID‐19 pandemic aiding the continued spread of AMR.^11^ A significant concern is the unwarranted use of antibiotics in patients displaying COVID‐19 symptoms, despite antibiotics being ineffective against viruses. This widespread use of antibiotics may increase the selection pressure on resistant bacteria, thereby contributing to their spread within and between healthcare settings. Moreover, the interruption of critical health services due to the COVID‐19 outbreak could affect the supply and quality of antibiotics, diagnostic tools, and measures to prevent and control infections.^12^ This may result in the inappropriate use of antibiotics, delayed detection of resistant infections, and increased transmission of resistant pathogens. Similarly, the diversion of attention and resources away from AMR as a priority issue at the national and global levels may be an additional challenge posed by the COVID‐19 pandemic.^5^

LMICs face a number of challenges when implementing effective AMR strategies, including limited surveillance capabilities, weak regulatory frameworks, limited access to quality‐assured diagnostics and antibiotics, a high prevalence of infectious diseases and comorbidities, insufficient infection prevention and control procedures, a lack of awareness and education among health professionals and communities, and socioeconomic factors that affect antibiotic demand and use.^4^, ^13^, ^14^ Moreover, LMICs might be less equipped to deal with the effects of AMR on their economies and healthcare systems.

No doubt, there is a significant knowledge gap in understanding the complex interplay between COVID‐19 and AMR in LMICs, highlighting the need for continuous studies, antimicrobial stewardship training, antibiotic awareness education, and AMR legislation. In this article, we explore the complex challenges presented by the coexistence of the COVID‐19 and AMR pandemics in LMICs. Through literature review, we offer potential solutions that can be adopted by LMICs to address both pandemics holistically. Our aim is to raise public awareness about the pressing need for LMICs to address both COVID‐19 and AMR, and to provide a framework for policymakers and stakeholders to develop effective strategies for managing these dual crises.

## 2 ANTIBIOTIC RESISTANCE IN LMICs

AMR, a profound menace to global public health, presents an especially daunting challenge in LMICs where up to 80% of prevalent illnesses exhibit resistance to at least one commonly utilized antibiotic, underscoring the severity of the AMR problem in these vulnerable populations.^3^ As up to 50% of antibiotic prescriptions are unnecessary, there is a correlation between the overuse and abuse of antibiotics and the high degree of resistance.^15^ The emergence of bacterial strains that are resistant to antibiotics, which are more challenging to treat, is facilitated by this misuse of antibiotics.^16^

The escalating prevalence of AMR in LMICs is deeply concerning. A significant contributor to this issue is the scarcity of robust antimicrobial stewardship programs (ASPs) in these regions.^17^ Without effective ASPs, the misuse and overuse of antibiotics become more prevalent, leading to increased resistance. Illustrating the severity of the situation, recent studies from various regions have reported alarming data. Recent studies from various regions provide a stark illustration: over 75% of certain *Klebsiella pneumoniae* and *Escherichia coli* isolates in Malawi were ceftriaxone resistant,^18^ with alarming results also reported in Egypt, Nepal, Nigeria, Pakistan, and other countries.^18^ A research from India showed that close to 75% of *K. pneumoniae* samples resisted cefepime, and around half resisted carbapenem.^18^

A wider viewpoint from a WHO report indicates that in both Africa and South‐East Asia, multidrug resistant (MDR) bacteria are responsible for 45% of deaths. This report emphasizes the intense resistance of *K. pneumoniae* to third‐generation cephalosporins, which is linked to increased mortality rates in different areas.^19^ This pattern is also seen in the frequent isolation of methicillin‐resistant *Staphylococcus aureus* (MRSA) in healthcare environments throughout African countries, with incidence rates in Cameroon, for instance, climbing up to 72%.^20^ Similarly, a 2008 South African study further emphasizes this, with 57.1% of nosocomially acquired Enterobacteriaceae infections being MDR.^21^ These findings echo an alarming report from the Government of India, showing resistance to fluoroquinolones and third‐generation cephalosporins in over 70% of *K. pneumonia* isolates.^22^ Furthermore, a scoping review underscored the widespread concern about MDR Gram‐negative bacilli in ICU infections in LMICs, with particular emphasis on high resistance to third‐generation cephalosporins and carbapenems.^23^ These findings are corroborated by a systematic review and meta‐analysis by Costello et al.,^24^ linking longer durations and multiple courses of antibiotics to higher resistance rates in LMICs.

Regrettably, the troubling scenario is poised to deteriorate further due to the influence of COVID‐19 on the development and dissemination of AMR in LMICs. Currently, there's an ongoing effort to fully implement antimicrobial stewardship in eight African countries at national level to combat the spread of AMR.^25^ However, despite effort being made for the full implementation and incorporation of AMR stewardship programs in LMICs, there's still a paucity of information on the success of these programs and the impact of COVID‐19 in aggravating the AMR in LMICs.^18^

## 3 THE COVID‐19 PANDEMIC IN LMICs

During the initial phase of the COVID‐19 pandemic, the majority of the documented illness and deaths related to COVID‐19 took place in high‐income nations.^26^ The pandemic stands out as the most severe of the 21st century, leading to almost seven million fatalities to date and touching every part of the globe.^27^ The progression of the outbreak has been diverse, marked by several surges that impacted different countries and continents with differing death rates.^26^, ^27^

In LMICs, in comparison to other countries, countries like Peru, South Africa, Brazil, Vietnam, and India experienced high rate of deaths associated with COVID‐19.^28^ While the precise cause for the variation isn't fully understood, the limited extent of COVID‐19 testing, underreporting of cases, and the absence of sero‐surveys and tracking systems to monitor hospitalizations and deaths related to COVID‐19 are seen as key contributing elements.^29^

## 4 THE CHALLENGES THAT LMICs FACE IN RESPONDING TO THE PANDEMIC

Handling the COVID‐19 pandemic is particularly challenging for LMICs, given the significant economic and health resource issues it creates.^10^ In hospitals within LMICs that face a shortage of oxygen tanks, there was a notable rise in distress and death rates.^30^ Undeniably, for LMICs with vulnerable healthcare systems, having effective preparedness for outbreaks of this scale, like COVID‐19, is challenging.^31^, ^32^ These factors have led to the breakdown of the current health system at the cost of essential healthcare needs.^33^, ^34^ The second challenge arose from the system's inability to respond to the growing demand for intensive care support, particularly for individuals with comorbidities who required more complex medical attention.^33^ The third challenge was a significant shortage of personal protective equipment (PPE) for frontline healthcare workers, exacerbated by panic purchasing and stockpiling. Additionally, the availability of essential protective items like gloves, medical masks, respirators, goggles, face shields, gowns, and aprons are limited in these areas, which further impedes prompt control of COVID‐19. Fourth, many elderly individuals in LMICs don't have access to basic healthcare as services are sparse and ill‐equipped to offer appropriate care for older people during the outbreak. Lastly, high population density is prevalent in many LMICs. Therefore, social distancing guidelines were often overlooked due to the socio‐cultural, economic contexts, and the realities of the individuals in these communities.^10^

## 5 THE INTERPLAY BETWEEN AMR AND THE COVID‐19 PANDEMIC IN LMICs

The emergence of the COVID‐19 outbreak underscores a multifaceted relationship with AMR. Although the pandemic's influence on AMR is a global concern, its detrimental effects are magnified in LMICs. Contributing factors include a surge in antimicrobial consumption, overreliance on empiric therapy, prevalent misdiagnosis, interruptions in the antimicrobial supply chain, inadequate PPE, and a diversion of financial resources from AMR control to COVID‐19 response. This convergence presents a unique and pressing challenge for LMICs, further straining already fragile healthcare infrastructures.^4^, ^35^

## 6 INDISCRIMINATE AND INAPPROPRIATE ANTIBIOTIC USE DURING THE PANDEMIC

From the onset of the pandemic, the focus has primarily been on diagnosing and managing COVID‐19, while the effects on AMR have mostly been neglected.^36^ The management of COVID‐19 often includes the synergistic prophylactic of broad‐spectrum antimicrobials.^37^ Usage of antimicrobials is exponentially higher than in the pre‐COVID epoch, especially in LMIC,^4^, ^5^, ^12^ where these kinds of drugs are available over the counter. The management COVID‐19 patients with antiparasitic, antiviral, antibacterial, and anti‐inflammatory medications to prevent secondary infections may unavoidably result in additional problems, such as a worsening of AMR.^38^ Similarly, the multisymptoms characteristic of COVID‐19 coinciding with other infections such as tuberculosis (cough) and malaria (fever) also lead to misdiagnosis and misapplication of antimicrobials.^39^ A study from Vanuatu, a small LMIC situated in southwest Pacific, reported that there was an escalation in the usage of antibiotics during the COVID‐19 pandemic than before.^40^ Similarly in Pakistan, enormous increases in consumption of azithromycin have been reported.^41^ Further, the pecuniary shocks brought on by COVID‐19 regulations may make it more necessary for people in LMICs, where antimicrobial purchases are already prone to less scrutiny, to misuse them in an effort to cut healthcare costs.^39^

## 7 DISRUPTION OF ANTIBIOTIC SUPPLY CHAIN

Antimicrobial stockpile chains and the world production rate have both been interrupted by COVID‐19, which has had an impact on accessibility and resulted in variations to application patterns. Owing to the significant disruption in manufacturing countries, the COVID‐19 pandemic has affected the delivery of antibiotics to LMICs. This could result in the use of inferior or counterfeit antibiotics, further fueling resistance development.^39^ Moreover, access limitation also means that drug resistant infections can remain untreated, further complicating the AMR crisis.

## 8 THE USE OF TELEMEDICINE

The pandemic's prevention strategy such as lockdowns demonstrated the value of telemedicine. However, telemedicine has been shown to result in overprescription of antibiotics compared to routine clinical appointments, and therefore, the challenge of accurately assessing patients through this method necessitates the physician to go beyond clinical acumen to comprehend the microbial infection and its nature,^42^ which in turn intensifies the rate of AMR.

## 9 IN PROPER WASTE MANAGEMENT

Due to excessive use and an inadequate waste management system in LMICs,^5^ inappropriate PPE such as face masks and surgical gloves can be used, which in turn aids propagation and favors horizontal gene transfer between resistant and nonresistant organisms.^42^

## 10 DIVERSION OF RESOURCES, RESEARCH, AND SERVICE TO COVID‐19 RESPONSE

The pandemic has not only overloaded the healthcare and laboratory systems, it has also disrupted a number of industries and research facilities, which include pharmaceuticals, which concentrated all of their research on developing novel COVID‐19 diagnostic tools, medications, and vaccines whilst also ignoring other research on infectious diseases,^43^ as well as diverted funding, manpower, partnerships, and supplies.^44^ During the pandemic, many collaborative research efforts and AMR‐related initiatives, encompassing national AMR surveillance programs and clinical trials, were put on hold. These interruptions have deeply affected LMICs, which are particularly susceptible to public health emergencies.^45^

ASPs, designed to promote judicious antibiotic use, deter misuse, and reduce the risk of ensuing resistance,^46^ have regrettably experienced setbacks. Resources meant for these operations have diverted to the management of the COVID‐19 pandemic.^39^ In the same vain, it has been reported that the availability of laboratory machines, reagents, and consumables for AMR has decreased in response to COVID‐19.^44^ The development of vaccines and supply networks had also been hampered by strict regulations and lockdown measures. The vaccine shortages for infectious diseases like measles, TB, and others will have devastating effects, as is foreseeable.^43^ Most of the antimicrobials used in LMICs are imported from developed countries; therefore, restrictions on local and international conveyance during this pandemic may directly affect the supply of AMR response consumables for ASP.^44^ The COVID‐19 pandemic may also interrupt standard healthcare practices pertaining to antibiotic stewardship and infection prevention and control, such as the isolation and management of individuals who have been exposed to or infected with AMR bacteria, such as those with MDR‐TB infections.^47^

## 11 INCREASED RISK OF HEALTHCARE‐ASSOCIATED INFECTIONS (HAIs) DUE TO COVID‐19 TRANSMISSION

HAIs occur as a result of a patient's admission to a healthcare facility.^48^ About 75% of these infections occur in LMICs,^49^ and it has been attributed to an increase in length of hospitalization, rate of illnesses and deaths, severity, as well as healthcare outlays.^48^ Generally, COVID‐19 has submerged healthcare facilities in huge hassles, which emphasize the crucial importance of control and prevention of infection practices in protecting both the community and healthcare personnel.^48^ The chances of the emergence of AMR has been established to rise with the in‐hospital spread of microbes that are MDR.^42^ In light of this, developing countries are more likely to experience outbreaks and emergences due to the problem than developed countries.^42^, ^48^

## 12 THE POTENTIAL LONG‐TERM CONSEQUENCES OF THE DUAL CRISIS ON PUBLIC HEALTH IN LMICs

In LMICs, the dual problem of COVID‐19 and AMR has the potential to have serious and enduring effects on public health. The COVID‐19 pandemic, by catalyzing the escalated utilization of antibiotics and other antimicrobial agents, has potentially exacerbated the proliferation and emergence of antibiotic‐resistant bacterial strains, thereby intensifying the AMR conundrum. Due to their lack of resources, inadequate health systems, and high prevalence of infectious diseases, LMICs are especially susceptible to the twin COVID‐19 and AMR dilemma. Increased mortality and morbidity brought on by illnesses that are more difficult to treat and avoid could be one effect of the dual crises.^33^, ^50^ Similarly, extended hospital stays, intensive care, and additional therapies could result in an increase in healthcare expenses.^50^ Moreover, LMICs’ health systems might become overburdened and disrupted, which would lower access. Eventual loss of money, livelihoods, and education may equally increase poverty and inequality.^14^

Also, because of weak infection prevention and control procedures, low sanitation and hygiene standards, and environmental contamination, the dual crises may result in an increase in the transmission and spread of resistant bacteria.^5^, ^50^ Malnutrition, comorbidities, immunosuppression, and a lack of vaccination may increase susceptibility to resistant illnesses.^3^, ^50^ The situation might get worse if novel resistance mechanisms start to appear and spread among bacteria as a result of genetic exchange and horizontal gene transfer.^3^, ^5^ Furthermore, the twin crises may also lessen the efficacy and accessibility of both new and existing antimicrobials due to the emergence of resistance, irrational use, poor quality, and supply chain disruptions.^3^, ^5^ The realization of the Sustainable Development Goals and the Universal Health Coverage (UHC) objectives in LMICs might be profoundly hindered by these repercussions.

The interruption of immunization programs in LMICs as a direct consequence of COVID‐19 brings the risk of serious long‐term effects, such as the populace's vulnerability to other infectious diseases, especially in children, which may necessitate antimicrobial treatment.^51^ In LMICs, the dearth of PPE has engendered unsafe medical practices, such as the sharing or reuse of PPE, thereby elevating the risk of cross‐transmission. This phenomenon is frequently implicated in the proliferation of multidrug‐resistant organism outbreaks.^51^

## 13 RECOMMENDATIONS AND FUTURE DIRECTION

To tackle this dual challenge, enhancing infection prevention measures and fortifying antibiotic stewardship programs are imperative. Specifically, for LMICs, there must be a focus on achievable and sustainable goals that do not solely depend on external funds. To optimize antibiotic use, focused efforts must be placed on reducing unnecessary prescriptions, enhancing monitoring mechanisms for antibiotic usage, and aligning practices with WHO's Global Action Plan on Antimicrobial Resistance. The antibiotic stewardship programs must be strengthened.^17^ To accomplish this, it is vital to build mechanisms for monitoring and analyzing antibiotic usage, develop robust antibiotic policies and guidelines, and educate healthcare professionals on proper antibiotic use, considering specific challenges and existing resources within LMICs.

To curb the proliferation of infectious diseases and reduce the reliance on antimicrobial drugs, bolstering infection prevention measures like hand sanitation and environmental sanitation are of paramount importance.^12^ While the concept of accessing clean water, sanitation, and hygiene is recognized, the approach must focus on how LMICs can practically and sustainably attain these goals. For example, LMICs could invest in community‐driven sanitation projects that leverage local materials and expertise, or partner with regional organizations to implement water purification systems that can be maintained with existing resources and knowledge. These strategies prioritize local context and provide more feasible paths for long‐term sustainability. Furthermore, the integration of education and community engagement in water and sanitation projects can empower local populations to take ownership of these initiatives. Through collaborating with schools, local governments, and community leaders, LMICs can create a culture of awareness and responsibility around hygiene practices, thereby fostering a more sustainable approach to addressing these essential needs.

Considering the significant challenges associated with discovering new antimicrobials and alternatives, and the high costs involved, a shift towards supporting practical strategies is necessary for LMICs. This includes building regulatory frameworks that align with existing resources, fostering regional collaboration, and focusing on accessible therapeutic options. LMICs must build regulatory frameworks for medication approval and registration, provide incentives for research and development, and foster cooperation between academia, business, and the government to adapt existing therapeutic strategies rather than solely relying on the creation of novel therapies.^50^ This is necessary since new therapies such as phage therapy entails consideration of the long‐term planning, funding, and expertise. Similarly, adressing the dual crises requires strengthening healthcare infrastructure and systems, which includes expanding access to healthcare, increasing laboratory capacity, and hiring and educating more healthcare professionals.^12^, ^50^ To do this, LMICs must boost healthcare financing, enhance health information systems, and encourage cross‐sectoral cooperation across the environmental, agricultural, and health sectors.

However, LMICs frequently encounter structural obstacles to putting these initiatives into practice, such as a lack of funding, infrastructure, and technical know‐how. The focus must be on leveraging existing resources, national action plans, and aligning with international guidelines tailored to LMICs’ needs. As a result, international cooperation and finance are essential for helping LMICs deal with the dual problem.^13^ To bolster the capabilities of LMICs in the execution of recommended interventions, fortifying their proficiency in infectious disease prevention and control, mitigating the proliferation of resistant variants, and ensuring equitable and sustained healthcare access, global benefactors should proffer fiscal assistance, technical expertise, and facilitate the exchange of best practices. International collaboration and funding can help promote equity and sustainability in addressing the dual crisis and ensure a coordinated global response to this threat to global health. They can also make it easier for different countries and regions to share knowledge, best practices, and resources.

## 14 CONCLUSION

The confluence of the COVID‐19 pandemic and the escalating crisis of AMR presents significant and multifaceted challenges in LMICs. Factors contributing to this complex scenario include the augmented use of antimicrobials, overprescription of antibiotics, dependence on empiric therapy, frequent misdiagnosis, improper antimicrobial utilization, heightened exposure to antibiotic‐resistant pathogens, interruptions in antimicrobial supply chains and global production, inadequacies in PPE, financial strains, and resource allocation away from antibiotic stewardship initiatives toward COVID‐19 responses. Collectively, these dynamics disproportionately accelerate AMR in LMICs. Amidst the conjoined pressures of increased antibiotic utilization and disrupted health infrastructures, the synergistic crises of COVID‐19 and AMR may portend a durable and adverse effect on public health within these regions. Increased mortality, morbidity, medical costs, and poverty could result from this. Addressing the dual crises of COVID‐19 and AMR in LMICs requires a targeted and sustainable approach. This includes enhancing infection control practices, reducing unnecessary antibiotic prescriptions, focusing on practical solutions for clean water and sanitation, and fostering regional collaboration for new therapeutic strategies. Strengthening healthcare infrastructure, aligning with existing resources, and leveraging local expertise are vital to achieving lasting impact in the face of these complex challenges.

## AUTHOR CONTRIBUTIONS

**Bashar Haruna Gulumbe**: Conceptualization; Writing—original draft; Writing—review & editing. **Muhammed Rabiu Sahal**: Conceptualization; Writing—original draft; Writing—review & editing. **Abdulrakib Abdulrahim**: Writing—original draft; Writing—review & editing. **Abdullahi Adamu Faggo**: Writing—original draft; Writing—review & editing. **Zaharadeen Muhammad Yusuf**: Writing—original draft; Writing—review & editing. **Kabir Hassan Sambo**: Writing—original draft; Writing—review & editing. **Nazeef Idris Usman**: Writing—original draft; Writing—review & editing. **Musbahu Abdullahi Bagwai**: Writing—original draft; Writing—review & editing. **Wada Nafiu Muhammad**: Writing—original draft; Writing—review & editing. **Aliyu Adamu**: Writing—original draft; Writing—review & editing. **Uzairu Aminu**: Writing—original draft; Writing—review & editing. **Munkaila Tirmizhi Abubakar**: Writing—original draft; Writing—review & editing. **Kadai Alhaji Lawan**: Conceptualization; Writing—original draft; Writing—review & editing. All authors have read and approved the final version of the manuscript.

## CONFLICT OF INTEREST STATEMENT

The authors declare no conflicts of interest.

## TRANSPARENCY STATEMENT

The lead author Kadai Alhaji Lawan affirms that this manuscript is an honest, accurate, and transparent account of the study being reported; that no important aspects of the study have been omitted; and that any discrepancies from the study as planned (and, if relevant, registered) have been explained.

## Data Availability

Not applicable. Kadai Alhaji Lawan author had full access to all of the data in this study and takes complete responsibility for the integrity of the data and the accuracy of the data analysis.
